# Molecular mechanism analysis of *LdHSFB2a* in lily thermotolerance

**DOI:** 10.1007/s44154-025-00234-9

**Published:** 2025-07-01

**Authors:** Ting Li, Sujuan Xu, Yinyi Zhang, Liping Ding, Ze Wu, Nianjun Teng

**Affiliations:** 1 Key Laboratory of Landscaping, Ministry of Agriculture and Rural Affairs, Key Laboratory of Biology of Ornamental Plants in East China, National Forestry and Grassland Administration, College of Horticulture, Nanjing Agricultural University, Nanjing, 210095 China; 2 Nanjing Agricultural University-Nanjing Oriole Island Modern Agricultural Development Co., Ltd., Lily Science and Technology Backyard Qixia of Jiangsu/Jiangsu Graduate Workstation, Nanjing, 210043 China

**Keywords:** Lily, LdHSFB2a, Heat tolerance, HSF

## Abstract

Heat stress (HS) is a major environmental stress that inhibits plant growth and development. Plants have evolved various mechanisms to cope with heat stress, a key one being the HSF-HSP (Heat stress transcription factor-Heat shock protein) signaling pathway. HSFs can be divided into three classes: A, B, and C. In this study, we report the identification and functional characterization of a specific B2 member *LdHSFB2a* in *Lilium davidii* var. *unicolor*. RT-qPCR (Real-time Quantitative Polymerase Chain Reaction) analyses indicated that *LdHSFB2a* was highly expressed in HS-exposed leaves. LdHSFB2a was localized in the nucleus, consistent with the characterization of transcription factors. In contrast to other HSFBs, LdHSFB2a did not contain the typical B3 repression domain but exhibited transcriptional repression activity in yeast and plant cells. Transient overexpression and virus-induced gene silencing (VIGS) of *LdHSFB2a* in lily petals suggested that LdHSFB2a functions positively in lily thermotolerance. Consistent with the implication of *LdHSFB2a* function in thermotolerance, further analysis revealed that the expression levels of *HSFA1*, *HSFA2*, and *MBF1c* were increased as *LdHSFB2a* was overexpressed but reduced as *LdHSFB2a* was silenced. Furthermore, LdHSFB2a bound to the promoters of *HSFA3 A*, *WRKY33*, *CAT2*, and *GLOS1*. And *LdHSFB2a* overexpression and silencing enhanced and reduced their expressions, respectively. Therefore, we speculated that LdHSFB2a may be a coactivator that interacts with transcriptional activators to promote thermotolerance in lily by enhancing the expression of heat-responsive genes such as *HSFA3 A*, *WRKY33*, *CAT2*, and *GLOS1*.

**Supplementary Information:**

The online version contains supplementary material available at 10.1007/s44154-025-00234-9.

## Introduction

In recent years, rising temperatures have increased the frequency of extreme weather events. Studies have shown that high temperatures can negatively impact plant developmental processes, including photosynthesis, pollen vitality, cell division and differentiation, and seed formation (Hasanuzzaman et al. 2013; Jung et al. 2023; Zhang et al. 2022). As sessile organisms, plants have evolved various mechanisms to perceive external environmental signals and respond to biotic and abiotic stresses by modulating their morphology and gene expression (Liu et al. 2023c; Ding et al. 2020). Heat stress transcription factors (HSFs) play an important role in regulating the heat stress response in plants (Wu et al. 2022a, 2024a). HSFs can specifically bind to the conserved heat stress element (HSE) in the promoter region of *HSPs* (*Heat shock proteins*), induce rapid accumulation of HSPs, and recruit other transcription factors to form complexes to jointly improve plant heat stress tolerance (Ohama et al. 2016; Wu et al.2024b).

Lily (*Lilium* spp.) is an important horticultural crop with large, beautiful, fragrant flowers that substantially contribute to its commercial value (Zhang et al. 2023b). The optimal temperature for growing the main lily cultivars ranges from 16 ℃ to 24 ℃; temperatures beyond 30 ℃ will markedly affect flower quality and production (Lan et al. 2021; Yi et al. 2012). In China, temperatures often exceed 35 ℃ in summer, which severely decreases bulb and cut flower quality, ultimately restricting annual production. And studies investigating heat tolerance in lily remain limited and focused on class-A HSFs. Transcriptome analysis of *Lilium longiflorum* ‘White heaven’ showed that *HSFs* and *sHSPs* (*small Heat shock proteins*) reacted rapidly and strongly to heat stress and that differentially expressed genes of the ROS pathway were substantially induced. Further analysis revealed that silencing the heat-responsive marker gene *LlHSFA2* through BSMV-VIGS significantly reduced heat tolerance in lily plants. The expression of *sHSPs* was downregulated, while expression of *APX* genes (encoding major enzymes in the ROS scavenging system) was increased, indicating that *sHSPs* are pivotal in the crosstalk between the HSF-HSP and ROS pathways during the heat stress response of lily (Zhou et al. 2022; Diogo-Jr et al. 2023). Moreover, the HD-Zip I gene *LlHB16* improved lily and Arabidopsis thermotolerance by directly activating *HSFA2* and *MBF1c* expression in both plants but delayed flowering and ABA insensitivity in Arabidopsis, suggesting that LlHB16 collaboratively regulates thermotolerance through linking the basal heat-responsive pathway and ABA signal (Wu et al. 2022b). In Arabidopsis, *HSFA3* can be induced by drought and heat stresses and is an important component of HS memory (Schramm et al. 2008). We have previously established that at least two homologous genes, *LlHSFA3 A* and *LlHSFA3B*, had the same protein structure and exhibited similar expression patterns under HS. However, they played different roles in the heat stress response regulatory network of lily. LlHSFA3 A functioned positively in the early stages of HS by activating the expression of heat-related genes, while LlHSFA3B inhibited the expression of heat-related genes and played a negative role under long-term HS conditions (Wu et al. 2019). LlHSFB1 is a positive regulator of lily thermotolerance and can be directly trans-activated by the AP2/ERF member LlERF012 (Li et al. 2024). Nevertheless, the function and regulatory network of class-B HSFs in lily remain largely elusive.

In this study, *Lilium davidii* var. *unicolor* was selected for its ornamental value, edible bulbs, and poor heat tolerance (Liu et al. 2023a; Xu et al. 2024). An HSF from class B, *LdHSFB2a*, was identified from *Lilium davidii* var. *unicolor* and was induced via HS. LdHSFB2a exhibited transcriptional repression activity and was localized in the nucleus. Transient overexpression of *LdHSFB2a* in lily petal disks showed a moderate decrease in thermo-sensitivity compared to the control. Physiological indices such as DAB staining, relative ion leakage, MDA and H_2_O_2_ contents, and catalase activity were consistent with the phenotype of petals. Further analysis illustrated that *HSFA1*, *HSFA2*, and *MBF1c* expression were enhanced by *LdHSFB2a* expression, possibly contributing to the elevated thermotolerance. Conversely, silencing of *LdHSFB2a* via a VIGS assay resulted in decreased thermotolerance. Yeast one-hybrid assays validated that LdHSFB2a could bind to *HSFA3 A*, *WRKY33*, *CAT2*, and *GLOS1* promoters and positively regulate their expressions, which might contribute to the positive function of LdHSFB2a in lily heat stress response. We suggested that LdHSFB2a participates in lily thermotolerance through various regulatory pathways, including the HSF-HSP and ROS pathways, but the deeper mechanism needs further exploration.

## Results

### *LdHSFB2a* is a heat-inducible class B-HSF member

**Fig. 1:**
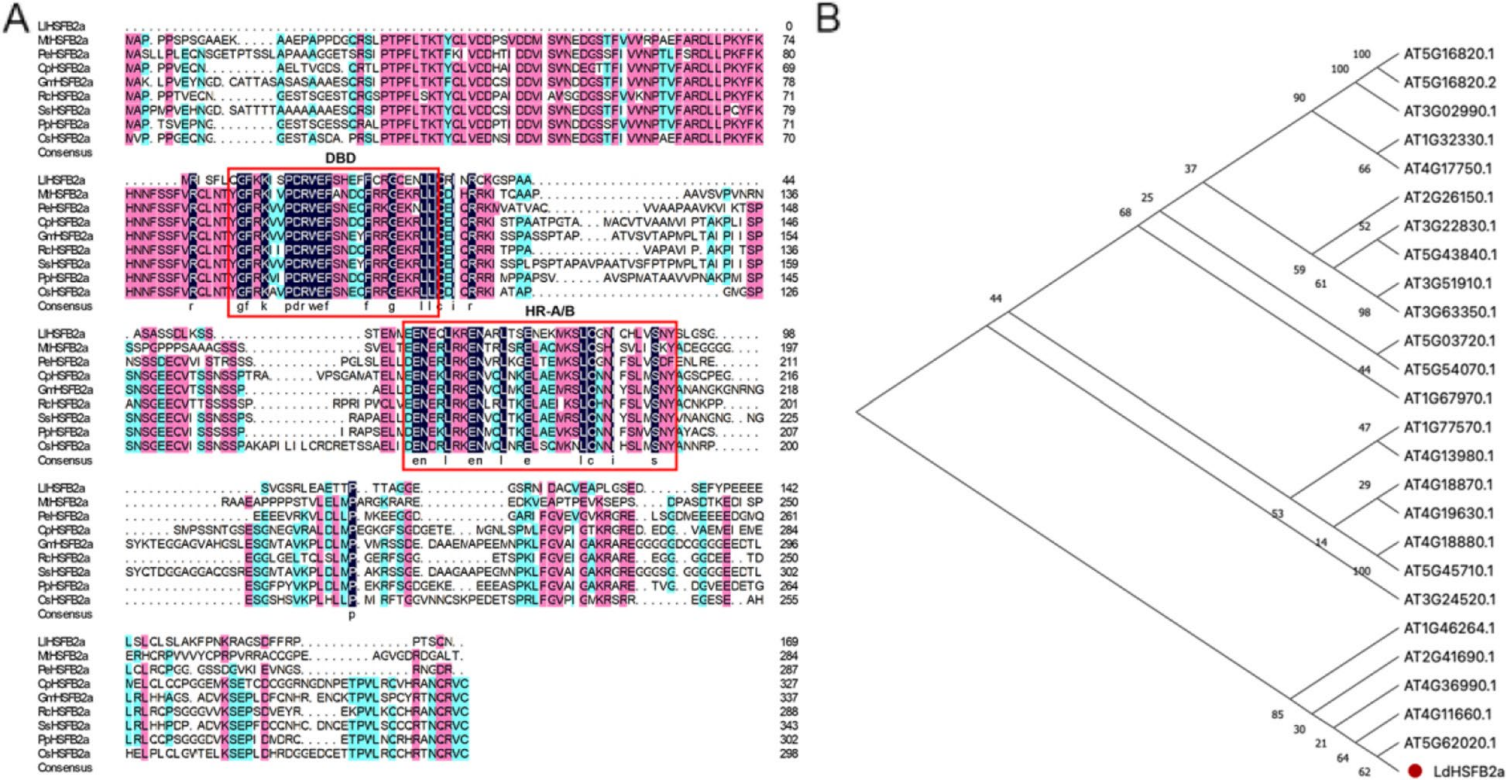
Multiple alignment of LdHSFB2a with amino acid sequences and phylogenetic analysis of LdHSFB2a and HSF members of Arabidopsis. **A** Multiple alignment of LdHSFB2a with those of homologous proteins known from other plant species. The conserved domains are labled with red boxes. DBD, DNA binding domain; HR-A/B, heptad repeats of hydrophobic amino acid residues. MtHSFB2a (*Musa troglodytarum*), PeHSFB2a (*Populus euphratica*); CpHSFB2a (*Carica papaya*), GmHSFB2a (*Glycine max*), RcHSFB2a (*Rosa chinensis*), SsHSFB2a (*Salix suchowensis*), PpHSFB2a (*Prunus persica*), CsHSFB2a (*Camellia sinensis*). **B** Phylogenetic analysis of LdHSFB2a and HSF members of Arabidopsis. The amino acid sequences were downloaded from the TAIR website (www.arabidopsis.org). The software tool MEGA X was used to reconstruct the evolutionary tree. Node values are percentages of bootstraps, generated with *n* = 1000 bootstrap replicates

**Fig. 2:**
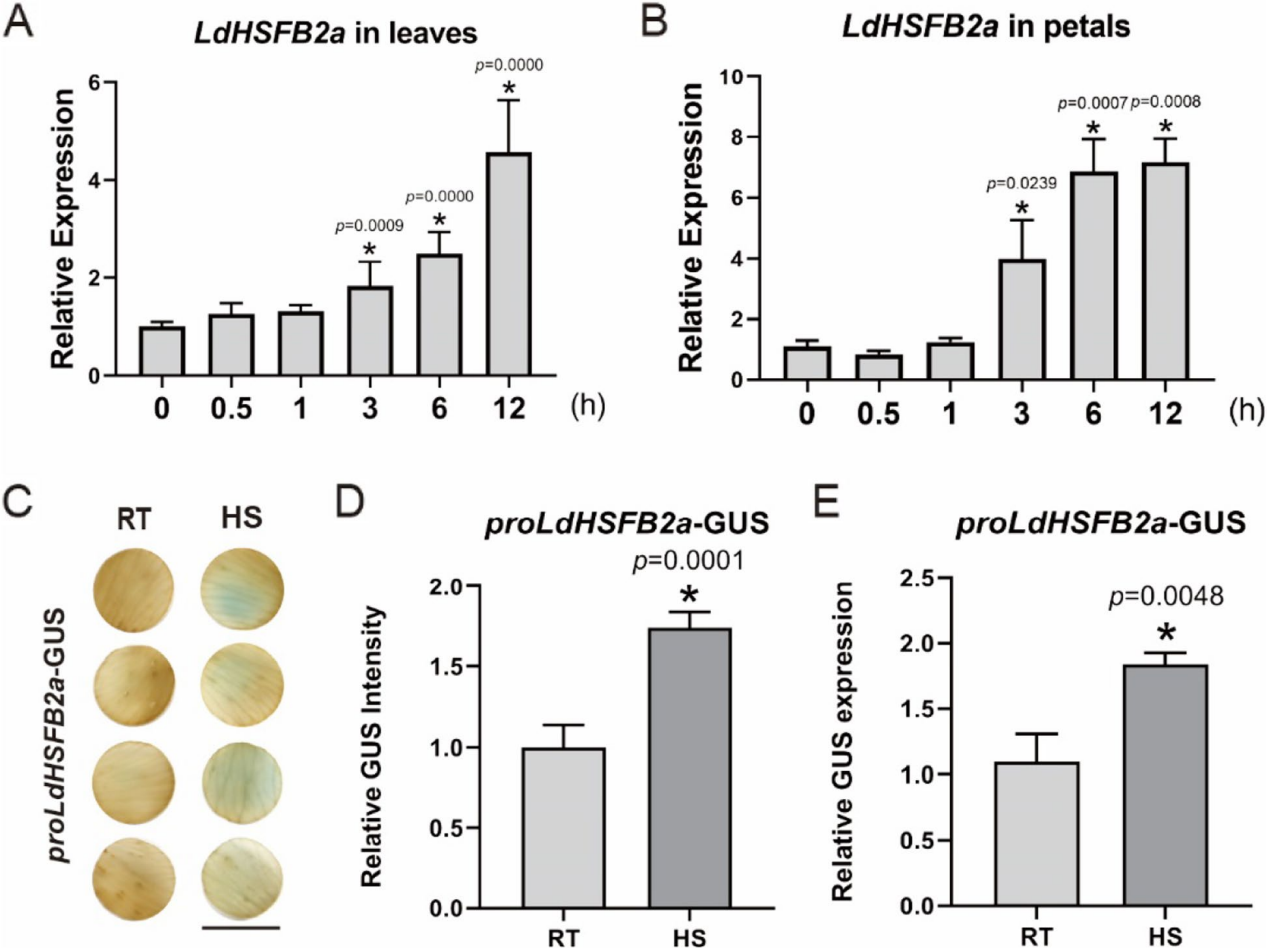
The expression levels and the promoter activity of *LdHSFB2a* under HS. **A** Detection of *LdHSFB2a* expression in lily leaves. Leaf samples were collected after applying to 37 °C in a standard culture room. The expression in lily leaves collected under 0-h HS treatment was used for normalization (set to 1). The *18S* rRNA of lily was used as the reference gene. Bars indicate the mean ± standard deviation (SD) (*n* = 3, Student's *t*-test, *p < 0.05). **B** Detection of *LdHSFB2a* expression in lily petals. Petal samples were collected after applying to 37 °C in a standard culture room. The expression in lily petals collected under 0-h HS treatment was used for normalization (set to 1). The *18S* rRNA of lily was used as the reference gene. Bars indicate the mean ± standard deviation (SD) (n = 3, Student's *t*-test, *p < 0.05). **C** The promoter activity of *LdHSFB2a* transiently transformed in lily petals RT (22 °C) and HS (37 °C, 3 h) conditions. Three independent experiments were performed, one representative picture was shown. Scale bar = 1 cm. **D** The GUS intensity in lily petal discs. The relative values are obtained by comparing with the control. Bars indicate the mean ± SD (*n* = 3, Student's *t*‐test, *p < 0.05). E GUS activity was quantified in transformed petals by measuring the expression of GUS gene through RT-qPCR. Lily *18S* rRNA was used as a normalization control. Relative expression levels were calculated using the 2.^−ΔΔCT^ method. Data are the mean ± SD of three independent experiments (*n* = 3, Student's *t*‐test, *p < 0.05)

*LdHSFB2a* was cloned from *Lilium davidii* var. *unicolor* leaves to explore its biological characteristics and functions. The ORF of *LdHSFB2a* was 510 bp, encoding a 169-amino acid peptide with a predicted molecular mass of 18.7 kD and an isoelectric point of 5.21. BLAST analysis using NCBI and TAIR databases revealed that LdHSFB2a belonged to the heat stress transcription family group B and had the highest homology with HSFB2a from other plant species. Amino acid sequence alignment indicated that LdHSFB2a contains DBD and HR-A/B domains, sharing similarity with other known HSFB2a members, but lacks the B3 repression domain (Fig. 1A). We used the MEGA X software to further explore the phylogenetic relationship between LdHSFB2a and members of the HSF family from Arabidopsis. The resulting phylogenetic tree illustrated that LdHSFB2a is the closest homolog of At5G62020.1 (AtHSFB2a; Fig. 1B). RT-qPCR analysis showed that the expression level of *LdHSFB2a* did not noticeably change at the initial HS stage (0—1 h) in lily leaves and petals. However, *LdHSFB2a* expression exhibited statistically significant increase at 3 h under HS, peaking at 12 h under HS (Fig. 2A and B). The *LdHSFB2a* promoter was isolated from *Lilium davidii* var. *unicolor* and fused with the *GUS* reporter gene to test its response to HS. The *LdHSFB2a* promoter showed heat-inducible GUS signals, with the results of GUS staining intensity aligning with those of the GUS expression analysis (Fig. 2C-E). Consequently, we speculated that high temperatures could activate *LdHSFB2a* expression.

### LdHSFB2a is a nuclear-positioned protein and exhibits transcriptional repression activity

**Fig. 3:**
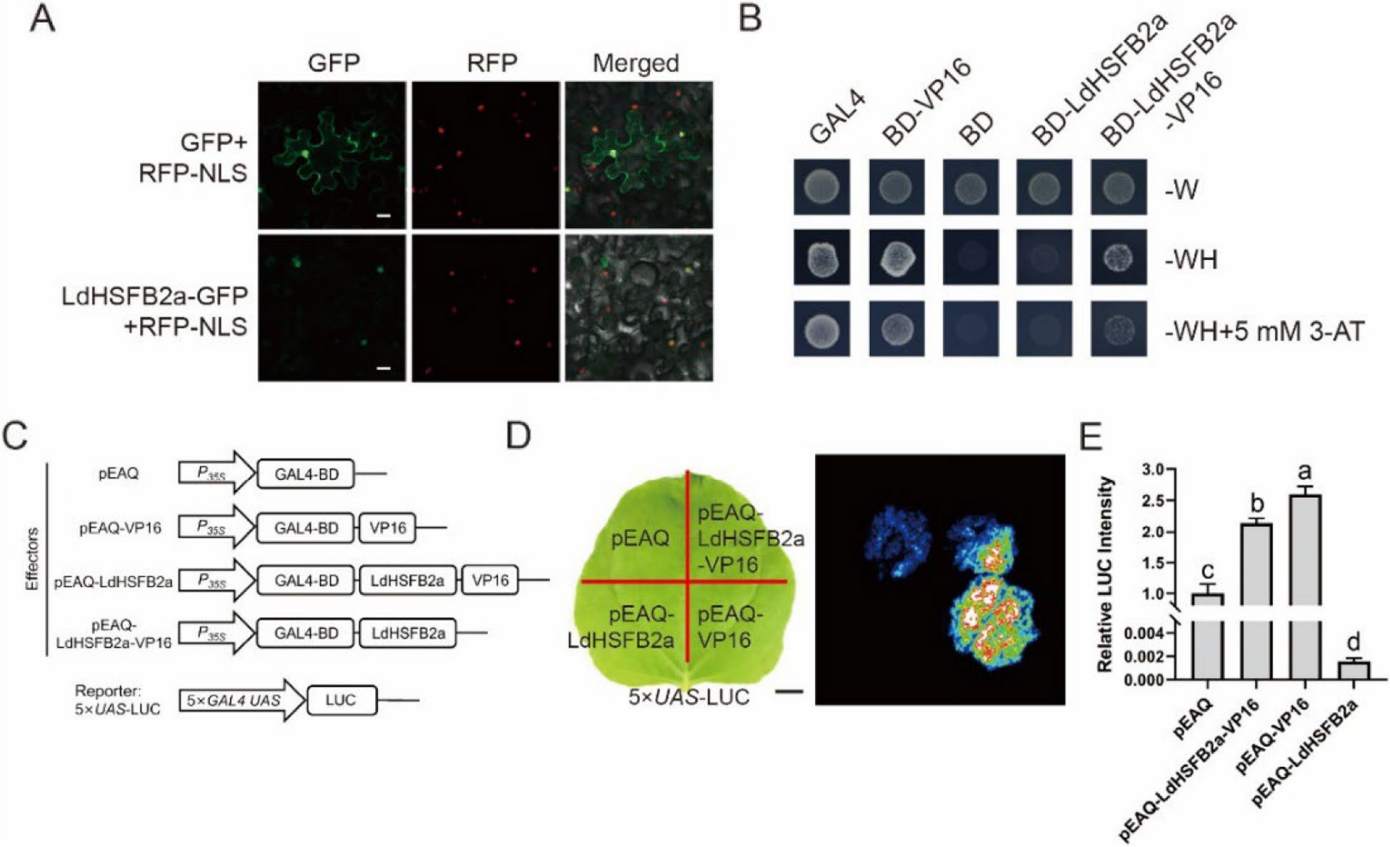
Subcellular localization and transcriptional activity assay of LdHSFB2a **A** Subcellular localization of LdHSFB2a protein in tobacco leaves. The empty GFP vector served as the negative control. The RFP-NLS used as a nucleus marker. Three experiments were performed, and one representative image is shown. Scale bar = 50 μm. **B** Transcriptional activity assay of LdHSFB2a in yeast cells. SD-Trp/-His medium (-WH) with 3-AT (3-amino- 1,2,4-triazole) was used to examine the transformants’ growth. GAL4 and BD-VP16 served as the positive controls; the empty vector BD served as the negative control. Three experiments were performed, and one representative image is shown. **C** Constructs used for the transcriptional activity assay in tobacco leaves. **D** The LUC signal in tobacco leaves. The empty vector pEAQ served as the negative control. The pEAQ-VP16 served as the positive control. Three experiments were performed, and one representative image is shown. Scale bar = 1 cm. E The LUC intensity in tobacco leaves. The LUC ratio of empty vector (pEAQ) and *UAS*-LUC combination was used for normalization (set to 1). Bars are means ± SD of three replicates with different letters indicating statistically significant difference (Student–Newman–Keuls test, *P* > 0.05)

Subcellular localization experiment was conducted to determine the location of LdHSFB2a in tobacco (*Nicotiana benthamiana*) cells. The LdHSFB2a-GFP fluorescence signal was mainly distributed in the nucleus and overlapped with the red signal of RFP-NLS, while the positive control (GFP) was dispersed throughout the nucleus and plasma membrane (Fig. 3A). Moreover, yeast one-hybrid assays showed that yeast transformed with the negative control pGBKT7 or pGBKT7-LdHSFB2a did not grow normally on SD/-Trp-His medium compared to yeast transformed with the positive control pGBKT7-GAL4 and pGBKT7-VP16. pGBKT7-LdHSFB2a-VP16 showed limited growth compared to pGBKT7-VP16, possibly repressed by 3-AT (Fig. 3B). To further test the transcriptional repression activity of LdHSFB2a, a GAL4/VP16‐UAS system was employed in tobacco leaves (Fig. 3C). The LUC signal of pEAQ‐LdHSFB2a-VP16 was substantially weakened compared to the positive control pEAQ‐VP16. The pEAQ‐LdHSFB2a LUC signal was also attenuated compared with that of the negative control pEAQ, suggesting that LdHSFB2a blocked the transactivation activity of VP16 and exhibited a repression ability in plant cells (Fig. 3D and E). These results showed that LdHSFB2a is a nuclear-positioned transcriptional repressor.

### Overexpression of *LdHSFB2a* increases heat tolerance in lily

**Fig. 4:**
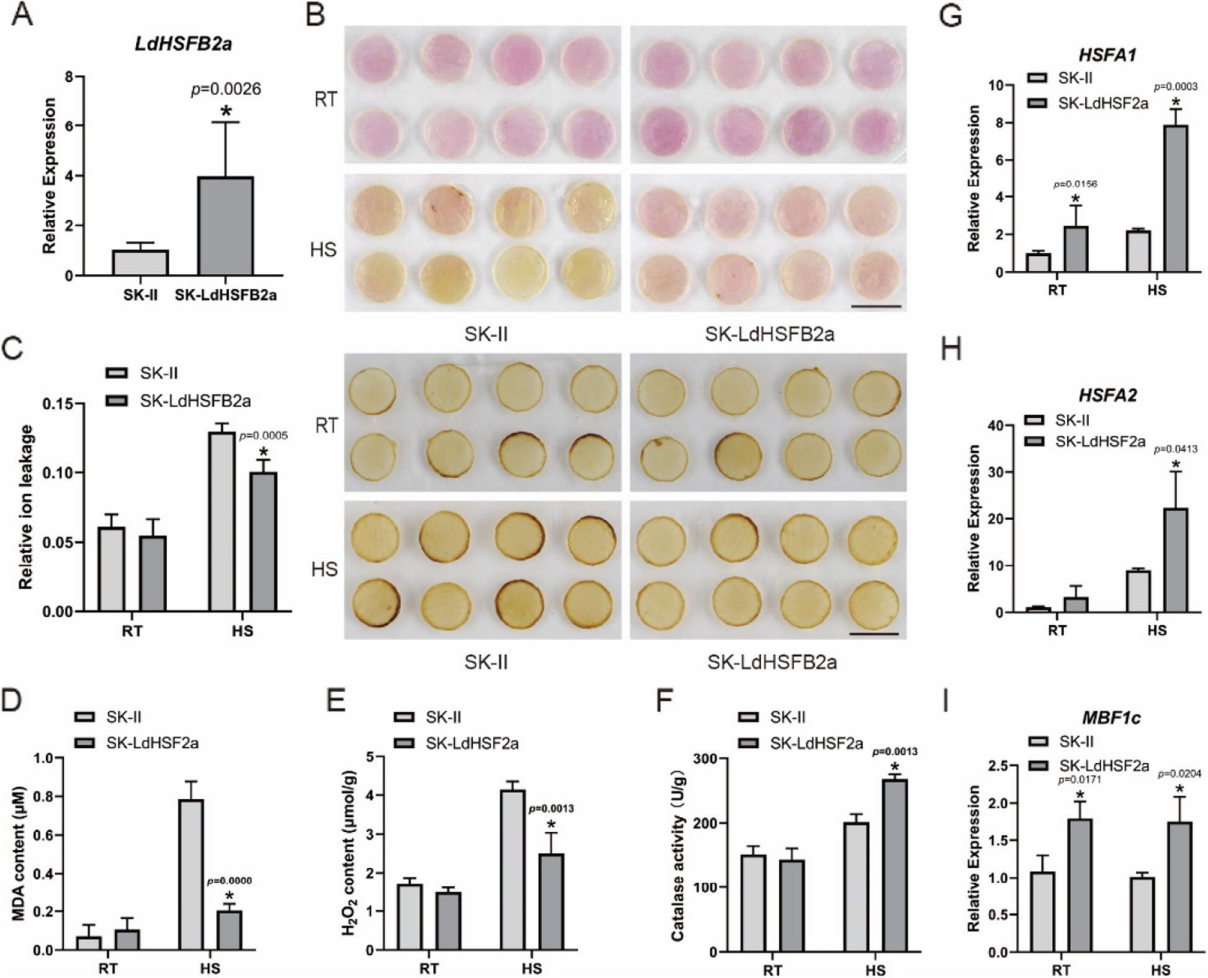
Transient overexpression of *LdHSFB2a* in lily petals. **A** The expression level of *LdHSFB2a* in transient-overexpressing lily petals. Lily *18S* rRNA was used as a normalization control. Relative expression levels were calculated using the 2.^−ΔΔCT^ method. Data are the mean ± SD of three independent experiments (Student's *t*‐test, *p < 0.05). **B** The phenotypes and DAB histochemical staining of *LdHSFB2a* transient-overexpression lily petals discs under normal conditions and after heat stress. Three experiments were performed, and one representative image is shown. Scale bar = 1 cm. **C** The effect of transient overexpression of *LdHSFB2a* in lily on relative ion leakage (%) under heat stress and room temperature. Data are the mean ± SD of three independent experiments (Student's *t*‐test, *p < 0.05). **D** The effect of transient overexpression of *LdHSFB2a* in lily on MDA (Malondialdehyde) content (μM) under heat stress and room temperature. Data are the mean ± SD of three independent experiments (Student's *t*‐test, *p < 0.05). **E** The effect of transient overexpression of *LdHSFB2a* in lily on H_2_O_2_ content (μmol/g) under heat stress and room temperature. Data are the mean ± SD of three independent experiments (Student's *t*‐test, *p < 0.05). **F** The effect of transient overexpression of *LdHSFB2a* in lily on catalase activity (U/g) under heat stress and room temperature. Data are the mean ± SD of three independent experiments (Student's *t*‐test, *p < 0.05). **G** The expression of *HSFA1* in *LdHSFB2a*‐overexpressing lily petals at RT (22 °C) and HS (40 °C, 3 h). Data are the mean ± SD of three independent experiments (Student's *t*‐test, *p < 0.05). **H** The expression of *HSFA2* in *LdHSFB2a*‐overexpressing lily petals at RT (22 °C) and HS (40 °C, 3 h). Data are the mean ± SD of three independent experiments (Student's *t*‐test, *p < 0.05). I The expression of *MBF1c* in *LdHSFB2a*‐overexpressing lily petals at RT (22 °C) and HS (40 °C, 3 h). Data are the mean ± SD of three independent experiments (Student's *t*‐test, *p < 0.05)

*LdHSFB2a* was overexpressed in lily petals to investigate its role in the plant heat stress response (Fig. 4A). No obvious phenotypic differences were noted between the control SK-II and SK-LdHSFB2a petals under normal conditions. However, under HS conditions, SK-II petals were more damaged than SK-LdHSFB2a petals. DAB staining showed that SK-II disks were deeper brown compared with SK-LdHSFB2a petals, suggesting that overexpressing *LdHSFB2a* results in less H_2_O_2_ accumulation (Fig. 4B). The conductivity of SK-II petals was notably higher than that of SK-LdHSFB2a petals under HS, indicating that cells in the control petals had more effluents and presented a higher degree of injury (Fig. 4C). Furthermore, MDA and H_2_O_2_ contents in SK-LdHSFB2a petals displayed a decreasing trend compared to heat-stressed SK-II petals, with no significant differences observed under normal conditions (Fig. 4D and E). Conversely, SK-LdHSFB2a petals subjected to HS exhibited higher catalase activity and had higher potential to scavenge ROS (Fig. 4F). Next, we performed RT-qPCR to measure the expression level of heat-related genes in SK-II and SK-LdHSFB2a petal disks. *LdHSFB2a* overexpression upregulated *HSFA1*, *HSFA2*, and *MBF1c* expressions under normal conditions and HS conditions (Fig. 4G-I). In summary, *LdHSFB2a* accumulation may improve lily thermotolerance under HS conditions.

### VIGS-mediated silencing of *LdHSFB2a* decreases heat tolerance in lily

**Fig. 5:**
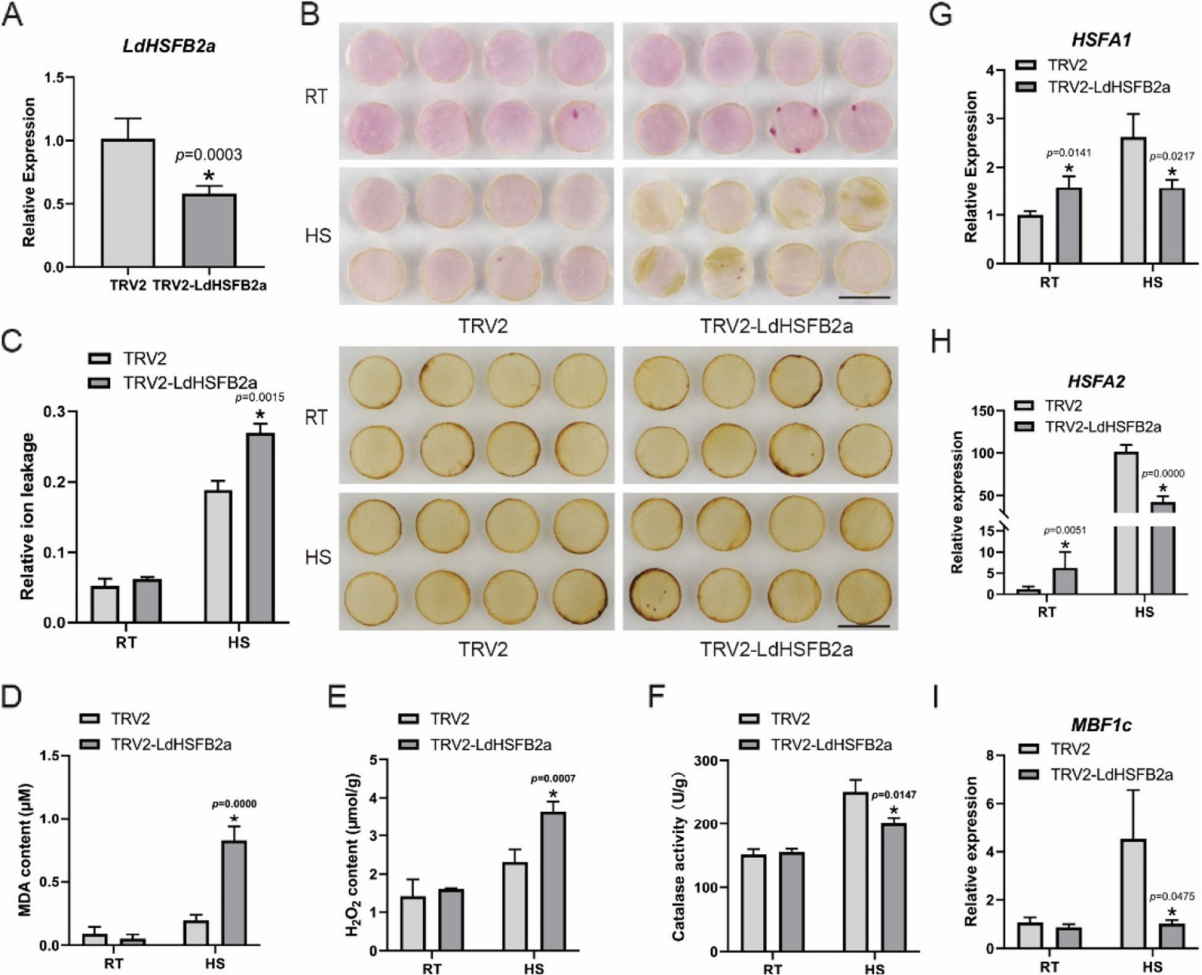
VIGS-mediated silencing of *LdHSFB2a* in lily petals **A** The expression level of *LdHSFB2a* in transient- silencing lily petals. Lily *18S* rRNA was used as a normalization control. Relative expression levels were calculated using the 2.^−ΔΔCT^ method. Data are the mean ± SD of three independent experiments (Student's *t*‐test, *p < 0.05). **B** The phenotypes and DAB histochemical staining of *LdHSFB2a* transient-silencing lily petals discs under normal conditions and after heat stress. Three experiments were performed, and one representative image is shown. Scale bar = 1 cm. **C** The effect of transient silencing of *LdHSFB2a* in lily on relative ion leakage (%) under heat stress and room temperature. Data are the mean ± SD of three independent experiments (Student's *t*‐test, *p < 0.05). **D** The effect of transient silencing of *LdHSFB2a* in lily on MDA (Malondialdehyde) content (μM) under heat stress and room temperature. Data are the mean ± SD of three independent experiments (Student's *t*‐test, *p < 0.05). **E** The effect of transient silencing of *LdHSFB2a* in lily on H_2_O_2_ content (μmol/g) under heat stress and room temperature. Data are the mean ± SD of three independent experiments (Student's *t*‐test, *p < 0.05). **F** The effect of transient silencing of *LdHSFB2a* in lily on catalase activity (U/g) under heat stress and room temperature. Data are the mean ± SD of three independent experiments (Student's *t*‐test, *p < 0.05). **G** The expression of *HSFA1* in *LdHSFB2a*‐silencing lily petals at RT (22 °C) and HS (40 °C, 3 h). Data are the mean ± SD of three independent experiments (Student's *t*‐test, *p < 0.05). **H** The expression of *HSFA2* in *LdHSFB2a*‐silencing lily petals at RT (22 °C) and HS (40 °C, 3 h). Data are the mean ± SD of three independent experiments (Student's *t*‐test, *p < 0.05). **I** The expression of *MBF1c* in *LdHSFB2a*‐silencing lily petals at RT (22 °C) and HS (40 °C, 3 h). Data are the mean ± SD of three independent experiments (Student's *t*‐test, *p < 0.05)

*Tobacco rattle virus* (TRV)-based virus-induced gene silencing (VIGS) is an effective and convenient tool for gene silencing and can be applied in various plant species (Bachan et al. 2012; Rössner et al. 2022). The VIGS method was employed using petal disks to silence *LdHSFB2a* in lily (Fig. 5A). No obvious difference was noted between the control TRV2 petals and *LdHSFB2a*-silencing petals under normal conditions. However, HS hastened the discoloration of TRV2-LdHSFB2a petals, resulting in a more brown-colored phenotype than the control. DAB staining also showed that TRV2-LdHSFB2a petals were deeper brown than TRV2 petals, indicating higher H_2_O_2_ accumulation in *LdHSFB2a-*silencing petals (Fig. 5B). Relative ion leakage was statistically significantly higher in TRV2-LdHSFB2a petals than in TRV2 petals, highlighting the lower cell survival rate in *LdHSFB2a-*silencing petal disks (Fig. 5C). Furthermore, MDA and H_2_O_2_ contents showed an increasing trend in TRV2-LdHSFB2a petals compared to TRV2 petals following HS, while no significant differences were observed under normal conditions (Fig. 5D and E). However, heat-stressed TRV2-LdHSFB2a petals exhibited lower catalase activity and had lower potential to scavenge ROS (Fig. 5F). RT-qPCR analyses were performed to measure the expression level of heat-related genes in TRV2 and TRV2-LdHSFB2a petal disks. Our findings suggested that *LdHSFB2a* silencing reduced *HSFA1*, *HSFA2*, and *MBF1c* expressions under HS conditions (Fig. 5G-I). Therefore, *LdHSFB2a* silencing may impair lily thermotolerance under HS conditions.

### LdHSFB2a binds the promoters of *HSFA3 A*, *WRKY33*, *CAT2* and *GOLS1*

**Fig. 6:**
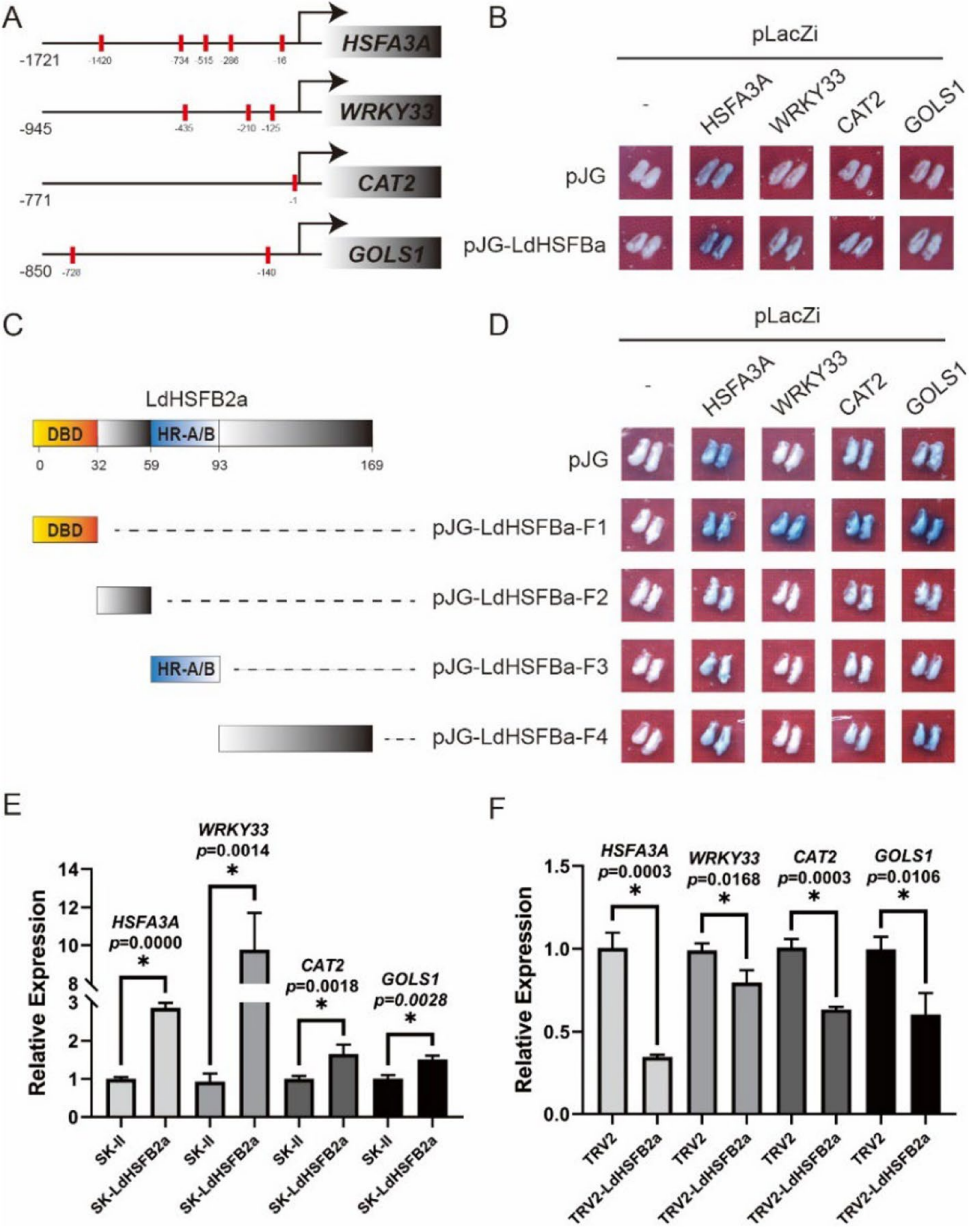
The interactions between LdHSFB2a and promoters of *HSFA3 A*, *WRKY33*, *CAT2* and *GOLS1.* **A** Diagram of *HSFA3 A*, *WRKY33*, *CAT2*, *GOLS1* promoters. The black lines indicate the tested promoter used for yeast one-hybrid (Y1H) assay. The red rectangle indicates the HSE. **B** Y1H validation of the interaction between LdHSFB2a and *HSFA3 A*, *WRKY33*, *CAT2*, *GOLS1* promoters by yeast cell growth on SD–Ura/–Trp deficient medium containing X‐gal (5‐bromo‐4‐chloro‐3‐indolyl β‐D‐galactopyranoside). Three independent experiments were performed, one representative picture was shown. **C** Diagram of LdHSFB2a. The coding region of LdHSFB2a was divided into four parts and tested in pairwise combinations to analyze interaction in Y1H assay. **D** Y1H validation of the interaction between LdHSFB2a-F1/F2/F3/F4 and *HSFA3 A*, *WRKY33*, *CAT2*, *GOLS1* promoters by yeast cell growth on SD–Ura/–Trp deficient medium containing X‐gal (5‐bromo‐4‐chloro‐3‐indolyl β‐D‐galactopyranoside). Three independent experiments were performed, one representative picture was shown. **E** The expression level of *HSFA3 A*, *WRKY33*, *CAT2*, *GOLS1* in *LdHSFB2a*-overexpressing lily petal discs. Lily *18S* rRNA was used as a normalisation control. Relative expression levels were calculated using the 2^−ΔΔCT^ method. Data are the mean ± SD of three independent experiments (Student's *t*‐test, *p < 0.05). **F** The expression level of *HSFA3 A*, *WRKY33*, *CAT2*, *GOLS1* in *LdHSFB2a*-silencing lily petal discs. Lily *18S* rRNA was used as a normalisation control. Relative expression levels were calculated using the 2.^−ΔΔCT^ method. Data are the mean ± SD of three independent experiments (Student's *t*‐test, *p < 0.05)

To further explore the regulatory mechanism of LdHSFB2a in lily thermotolerance, several promoters were selected and analyzed to find HSEs based on our previous results (Wu et al. 2019; Ding et al. 2024). Notably, five HSEs were found in the *HSFA3 A* promoter, three in the *WRKY33* promoter, one in the *CAT2* promoter, and two in the *GOLS1* promoter, respectively (Fig. 6A). The results of a yeast-one hybrid (Y1H) assay validated that LdHSFB2a interacted with the full-length of *HSFA3 A*, *WRKY33*, *CAT2*, and *GOLS1* promoters (Fig. 6B). And then, to identify the key region in LdSHFB2a of the interactions, LdHSFB2a was truncated and fused to pJG vector according to its conserved domains (Fig. 6C). The Y1H assay showed that the DBD of LdHSFB2a enabled the interactions between LdHSFB2a and the promoters of *HSFA3 A*, *WRKY33*, *CAT2*, and *GOLS1* (Fig. 6D). Furthermore, *LdHSFB2a* overexpression increased the endogenous expression of *HSFA3 A*, *WRKY33*, *CAT2*, and *GOLS1*, while *LdHSFB2a* silencing repressed their expressions (Fig. 6E and F). Thus, LdHSFB2a bound to the promoters of *HSFA3 A*, *WRKY33*, *CAT2*, and *GOLS1* and activated their expressions. LdHSFB2a might also exert direct regulatory effects on *HSFA3 A*, *WRKY33*, *CAT2*, and *GOLS1* to play potential roles in lily thermotolerance.

### *LdHSFB2a* expression correlates linearly with thermotolerance in different lily cultivars

**Fig. 7:**
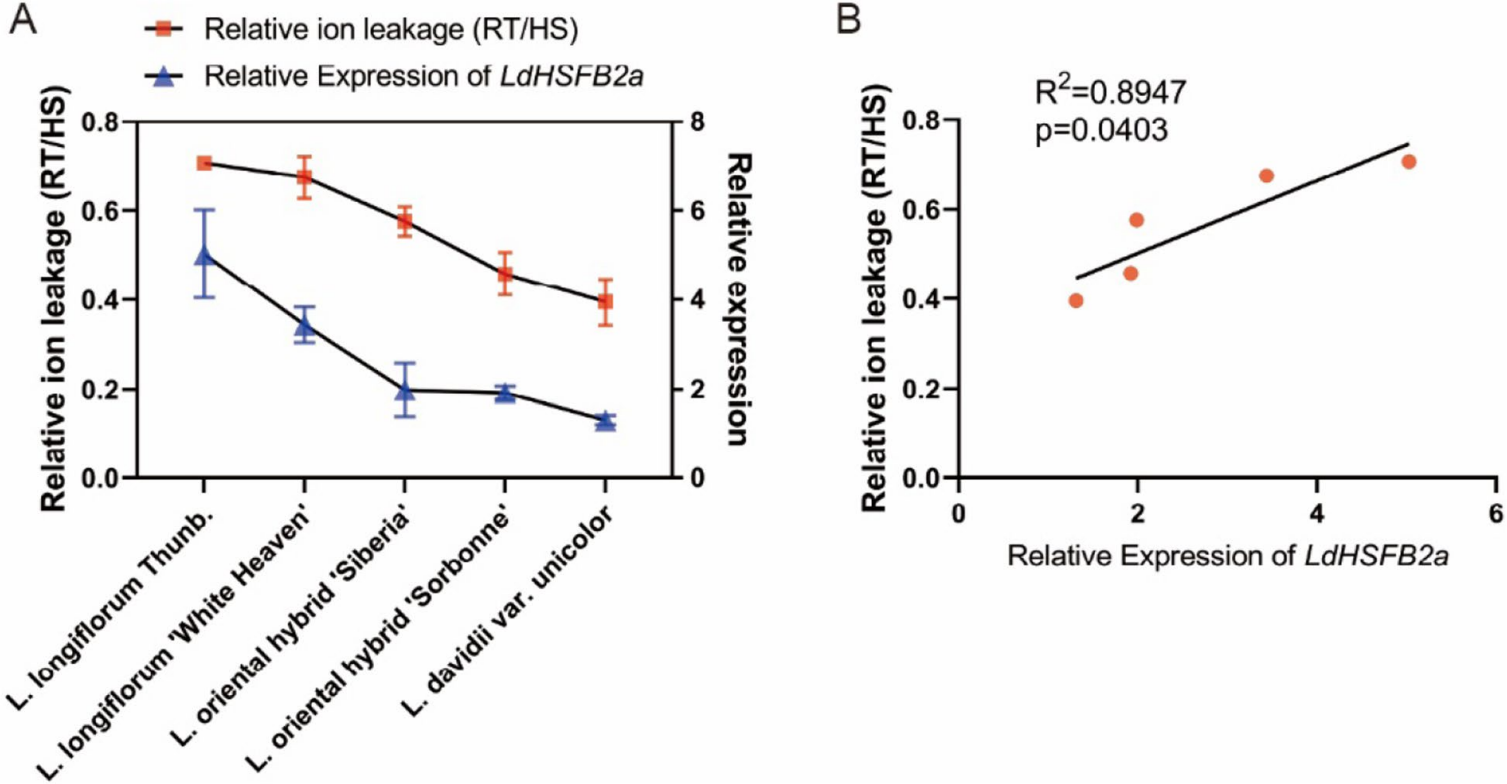
Pearson correlation analysis of *LdHSFB2a* expression and lily thermotolerance **A** Thermotolerance represented by the changing ratio of relative ion leakage before and after HS treatment (RT/HS) and *LdHSFB2a* expression in five lilies (HS, 37 °C/3 h). Lily *18S* rRNA was used as a normalisation control. Relative expression levels were calculated using the 2^−ΔΔCT^ method. Data are the mean ± SD of three independent experiments. **B** Pearson correlation analysis of *LdHSFB2a* expression and lily thermotolerance

To further explore the relationship between *LdHSFB2a* expression and lily thermotolerance, we analyzed *LdHSFB2a* expression in five different lily cultivars. *LdHSFB2a* expression in *L. longiflorum* Thunb. and *L. longiflorum* ‘White heaven’ were higher than those in the remaining three cultivars. *L. oriental* hybrid ‘Siberia’ and ‘Sorbonne’ exhibited a similar slight increase in expression levels following HS. However, the expression level in *L. davidii* var. *unicolour* was dramatically reduced following HS. This outcome might be related to previous findings that *L. davidii* var. *unicolour* showed a low heat tolerance through testing relative ion leakage in lily leaves before and after exposure to HS (Li et al. 2024; Fig. 7A). Next, the correlation between *LdHSFB2a* expression and lily thermotolerance was analyzed via Pearson correlation with a statistically significant difference (*R*^*2*^ = 0.8947, *p* = 0.0403; Fig. 7B). It was speculated that *LdHSFB2a* might play a significant role in lily heat stress response and that its expression could be a potential reference index for predicting lily thermotolerance.

## Discussion

We identified a class-B HSF, *LdHSFB2a*, from heat-stressed lily leaves (Fig. 1). As a class-B member, LdHSFB2a has no typical tetrapeptide -LFGV- in the carboxyl terminal, which was predicted to be a transcriptional repressor by interacting with another corepressor in the transcription machinery (Scharf et al. 2012). Therefore, it is worth exploring the transcriptional activity of LdHSFB2a due to its specificity. The results of the validation experiments in yeast AH109 and tobacco leaves showed that LdHSFB2a could block the strong transactivation of VP16 and exhibit transcriptional repression activity (Fig. 3B-E). The absence of the -LFGV- domain suggests that LdHSFB2a could have an atypical repression domain that remains unidentified.

HSFAs are mainly reported to play a positive regulatory role under HS (Fragkostefanakis et al. 2016; Friedrich et al. 2021). However, compared to HSFAs, the function of HSFBs is relatively complex. Studies have reported that the heat tolerance of *hsfb1* mutants is higher than that of wild-type plants, suggesting that *HSFB1* is a negative regulatory factor in the heat stress response (Fragkostefanakis et al. 2019). AtHSFB2a is ubiquitinated and degraded by the E3 ligase XBAT31, promoting downstream heat-stress-responsive genes (Zhang et al. 2024). In *Lactuca sativa*, heat stress increases the abundance of LsHSFB2 A- 1 proteins that upregulate *LsMADS55* expression and facilitate plant transitions from vegetative to reproductive growth (Ning et al. 2019). Silencing the homologous *Trifolium pratense* gene *TrHSFB2a* in Arabidopsis reduced relative ion leakage and MDA content, indicating that *TrHSFB2a* functioned negatively as well (Iqbal et al. 2022). Conversely, overexpressing *CarHSFB2* from chickpea in Arabidopsis notably promoted heat tolerance (Ma et al. 2015). High temperature induced the expression of *OsHSFB2b* in rice, which contributed to enhanced thermotolerance (Xiang et al. 2013). In our previous studies, LlHSFB1 was shown to be a positive regulator of lily thermotolerance. Therefore, class-B HSF members may play different roles in different plant species under heat stress. In the present study, heat stress elevated the expression of *LdHSFB2a* and the activity of its promoter (Fig. 2)*.* Transiently overexpressing *LdHSFB2a* in lily petal disks led to a decrease in relative ion leakage and H_2_O_2_ content and improved catalase activity compared to the control (Fig. 4A-F), while silencing *LdHSFB2a* via VIGS produced a contrasting effect (Fig. 5A-F). Thus, *LdHSFB2a* might play a positive role in lily thermotolerance. The regulation of the heat stress response by HSFs also involves other signal transduction pathways, such as ROS, ABA, and Ca^2+^ signaling (Suzuki et al. 2012; Torres and Berlanga 2023; Ding et al. 2021). With the help of cofactors, HSFs form transcription regulation complexes through phosphorylation or oligomerization, which activate the expression of *HSPs* and other genes (Gong et al. 2020; Xu and Xie 2023; Perrella et al. 2022; Luo et al. 2022). *MBF1c* is a key heat-related regulator that plays an important role in improving heat tolerance (Alavilli et al. 2017; Xiang et al. 2024). HSFAs also function positively under heat stress. HSFA2 is similar to HSFA1 in protein structure and function, and both are considered enhancers for plant cells to acquire heat tolerance (Gong et al. 2014). HSFA2 regulates gene expression under heat stress and oligomerizes with HSFA1 (Yoshida et al. 2011; Chan-Schaminet et al. 2009). To investigate the molecular mechanism by which *LdHSFB2a* may participate in lily thermotolerance, the expression levels of heat-related genes were measured in lily petals transiently transformed with *LdHSFB2a*. Under heat stress, the expression levels of *HSFA1*, *HSFA2*, and *MBF1c* in *LdHSFB2a*-expressed lily petals were higher than those in control petals, while the opposite trend was observed when *LdHSFB2a* was silenced via VIGS (Fig. 4G-I and Fig. 5G-I). Furthermore, Y1H assays showed that LdHSFB2a bound to the promoters of *HSFA3 A*, *WRKY33*, *CAT2*, and *GOLS1* and that the DBD of LdHSFB2a was necessary for the binding to occur (Fig. 6A-D). The accumulation of *LdHSFB2a* transcripts elevated their expressions, while a decrease in *LdHSFB2a* transcripts impaired their expressions (Fig. 6E and F). HSFA3 A was specifically required for physiological HS memory in Arabidopsis and short-term HS response in lily (Friedrich et al. 2021; Wu et al. 2019). WRKY33 was reported to play a central role in plant immune response (Chen et al. 2024; Liu et al. 2023b). And recent reports revealed that it also functioned in abiotic stress response, such as heat tolerance in *Lilium longiflorum*, Cadmium tolerance in *Arabidopsis* and salt tolerance in *Brassica napus* (Wu et al. 2023; Yan et al. 2024; Zhang et al. 2023a). In addition, CAT2 and GOLS1 are considered positive regulators of stress tolerance by scavenging excessive ROS and increasing antioxidative enzyme activities (Han et al. 2024; Li et al. 2023). Therefore, *LdHSFB2a* may take part in heat tolerance in lily through multiple regulatory pathways, e.g., the HSF-HSP and ROS pathways, by directly affecting *HSFA3 A*, *WRKY33*, *CAT2*, and *GOLS1* expression. As LdHSFB2a possesses transcriptional repression activity, LdHSFB2a may interact with other proteins to function as co-activators. The specific regulatory mechanism needs further investigation through genetic and molecular biology experiments.

## Conclusion

**Fig. 8:**
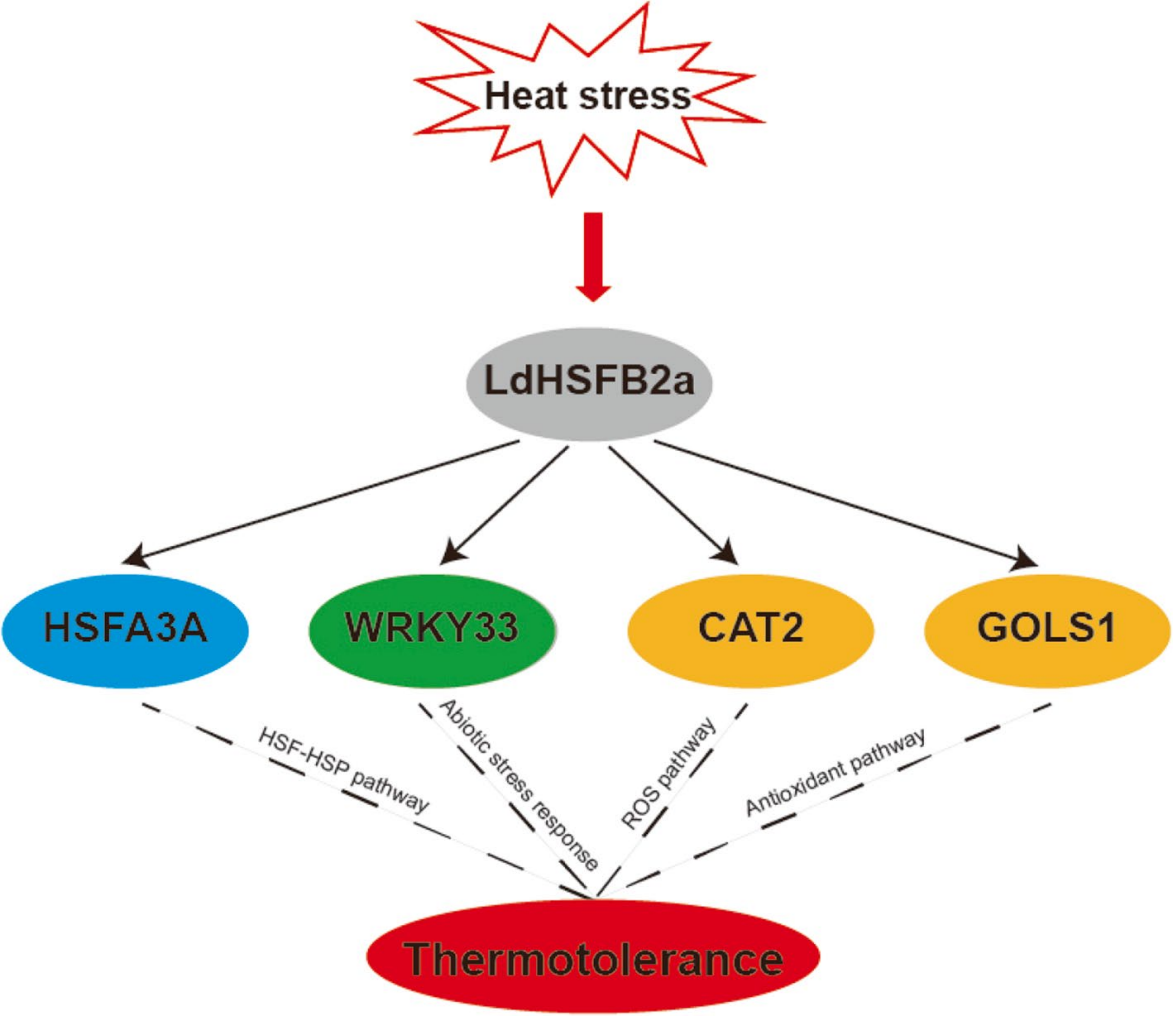
A schematic model of LdHSFB2a in heat stress regulatory network of lily LdHSFB2a can bind the promoters of *HSFA3 A* (HSF-HSP pathway), *WRKY33* (abiotic stress response), *CAT2* and *GLOS1* (ROS and antioxidant pathway), and regulate their expressions to increase lily thermotolerance

We identified LdHSFB2a as a specific, heat-inducible B2 member of the HSF family that positively participate in lily thermotolerance. *LdHSFB2a* expression was linearly correlated with heat tolerance in different lily cultivars. Moreover, LdHSFB2a could bind to the promoters of *HSFA3 A*, *WRKY33*, *CAT2*, and *GLOS1* and activate their expressions, implying it functioned in lily thermotolerance by mediating various regulatory pathways, such as the HSF-HSP and ROS pathways (Fig. 8). Despite these insights highlight the role of LdHSFB2a in plant heat stress response and the molecular breeding of heat‐tolerant lily cultivars, there still remain limitations, as all experiments were conducted under controlled laboratory conditions. In vitro systems cannot fully replicate the complex in vivo environment of field conditions. Future research will focus on developing stable *LdHSFB2a*-transgenic lily lines and evaluating their thermotolerance in field trials to validate their practical effectiveness.

## Methods and materials

### Plant materials and growth conditions

*Lilium davidii* var. *unicolor*, *Lilium longiflorum* Thunb., and lily cultivars ‘White heaven’ (*Lilium longiflorum*), ‘Siberia’ (*Lilium oriental*), ‘Sorbonne’ (*Lilium oriental*) were cultured on Murashige and Skoog (MS) medium and used for gene isolation, expression assays, and thermotolerance assays. *Lilium oriental* hybrid ‘Sorbonne’ buds were used as the heat-tolerance testing platform and planted in Baguazhou Lily Germplasm Resource Base, Nanjing Agricultural University. Tobacco (*Nicotiana benthamiana*) seeds were sterilized and sown on MS medium. After 3 d of vernalization at 4 ℃ in the dark, followed by 10 d of germination at 22 ℃ under light conditions, the seedlings were transferred to plastic pots containing vermiculite and peat soil (1:1). The plantlets grew under controlled conditions (22 ℃/16 ℃, 16-h light/8-h darkness).

### Isolation and sequence analysis of *LdHSFB2a*

*Lilium davidii* var. *unicolor* leaves were collected, and total RNA was extracted using Trizol Reagent (Invitrogen, USA). cDNA was synthesized using a reverse transcription system (R323–01, Vazyme, China). The *LdHSFB2a* ORF was obtained via PCR. The primers used for *LdHSFB2a* cloning are listed in Supplementary Table S1. Next, the prediction of conserved protein domains and phylogenetic analyses were conducted by collecting HSFB2a homologs from different plant species on the NCBI (https://www.ncbi.nlm.nih.gov/) website and HSF members from Arabidopsis on the TAIR (https://www.arabidopsis.org/) website. Multiple sequence alignments of amino acids were performed using DNAMAN and ClustalX 1.81. The phylogenetic tree was constructed based on the neighbor-joining method using MEGA X.

### Gene expression assay of *LdHSFB2a* in heat-stressed lily leaves and petals

Five similar-sized tissue-cultured lily seedlings were selected and exposed to HS at 37 ℃ for different durations. Each sample was set up with three biological replications. RNA was extracted from ‘White heaven’ leaves, as mentioned previously, using the *SteadyPure* Plant RNA Extraction Kit (AG21019; Accurate Biology), and the RNA quality and concentration were detected using a microspectrophotometer (Nano‐300; ALLSHENG) after removing the genomic DNA (Li et al. 2024). RT-qPCR was carried out with lily *18S* rRNA selected as the reference gene. The primers for RT-qPCR are listed in Supplementary Table S2.

### Promoter isolation and activity analysis

The promoter sequence of *LdHSFB2a* was obtained using HiTAIL‐PCR (Liu and Chen 2007). The *HSFA3 A*, *WRKY33*, *CAT2*, and *GOLS1* promoter sequences have been previously reported (Ding et al. 2024; Wu et al. 2019). The full length of the *LdHSFB2a* promoter (1264 bp) was inserted into the pCAMBIA1391-GUS vector, and then the constructed plasmid was transformed into *Agrobacterium tumefaciens* strain GV3101. The bacterial solution was centrifuged and resuspended in infiltration buffer (10 mM MgCl2, 10 mM MES, 200 mM acetosyringone). Lily petal disks were excised from the center of three inner petals from colored and unopened flower buds using a hole puncher. The petal disks were transferred to the prepared solution for 20 min in a vacuum. Transformed disks were washed with deionized water to remove excess bacteria. Next, the disks were spread on a semi-solid plate (0.4% agar). After co-culture in the dark for 1 d and under light conditions for 2 d, the plates were moved into a temperature-controlled incubator (DRP‐9082; Sumsung) at 37 °C for 3 h. Next, the disks were submerged into GUS staining buffer for 20 min in a vacuum. The disks were then incubated in GUS staining buffer at 37 °C for 12 h. The pigment in the disks was extracted using 75% ethanol. Relative GUS intensity was calculated using the ImageJ v1.8.0 software. All primers used for plasmid construction are listed in Supplementary Table S3.

### Subcellular localization and transcriptional activity analysis of LdHSFB2a

The *LdHSFB2a* ORF without the stop codon was inserted into the pCAMBIA1300 vector and fused with the GFP protein. All vectors were transformed into *A. tumefaciens* strain GV3101 separately. GFP-LdHSFB2a and the empty vector pCAMBIA1300-GFP were mixed separately with the nucleus marker RFP-NLS in a 3:1 ratio and infiltrated into tobacco leaves. After 2 d, the GFP signal was detected using a confocal laser-scanning microscope (LSM800, Zeiss, Germany) to define the location of the LdHSFB2a protein as previous reported (Xu et al. 2021).

The *LdHSFB2a* ORF was inserted into pGBKT7 to generate the BD-LdHSFB2a construct. To determine transcriptional activity, the recombinant plasmid, together with pGBKT7, GAL4, and BD-VP16, was transformed into yeast strain AH109 via the standard lithium acetate-polyethylene glycol-mediated transformation procedure and incubated on synthetic defined (SD) medium lacking Trp and His. The positive transformants were selected for spot assays on deficient SD medium lacking Trp and His. *LdHSFB2a* was constructed into pEAQ and pEAQ-VP16 vectors as effectors, and 5 × GAL4 UAS was cloned into the pGreenII 0800‐LUC vector as a reporter. pEAQ‐VP16 was used as a positive control, while empty pEAQ was used as the negative control. The recombinant plasmids were transformed into *A. tumefaciens* strain GV3101 (pSoup) and the corresponding mixed suspensions (effector:reporter = 1:1) were transformed into tobacco leaves using a syringe without a needle. The LUC signal was detected using a CCD camera, and LUC intensity was quantified using ImageJ v1.8.0. All primers used for plasmid construction are listed in Supplementary Table S3.

### Transient overexpression and VIGS-mediated silencing of *LdHSFB2a* in lily

*Agrobacterium tumefaciens* solutions containing the recombinant plasmid pGreenII 62-LdHSFB2a-SK and the control pGreenII 62-SK were cultured, collected, and resuspended in infiltration buffer (10 mM MgCl2, 200 mM acetosyringone, 10 mM MES; pH 5.6). Next, the inner petal of ‘Sorbonne’ was punched using a hole puncher (1 cm diameter). The petal disks were infected with the prepared resuspension under vacuum conditions. After infiltration, the petal disks were washed with deionized water three times and cultured on agar plates (0.4%) for 3 d. Disks were treated at 42 ℃ for 12 h to simulate HS conditions and immediately harvested to identify heat tolerance and determine relative ion leakage. Samples were collected at room temperature (RT) and exposed to HS at 42 ℃ for 3 h for analysis of relative gene expression level. The specific 210-bp fragment located at the carboxyl terminal of *LdHSFB2a* ORF was inserted into the pTRV2 vector to construct the pTRV2-LdHSFB2a recombinant plasmid. Following overnight culture, the strains were collected and resuspended to an OD_600_ of 1.0. pTRV1 was mixed with pTRV2 and pTRV2-LdHSFB2a at a ratio of 1:1, separately. Mixtures were placed in the dark for 3 h before agroinfiltration. The methods of injection and exposure to HS were the same as for transient overexpression. The disks for gene expression analysis were also sampled at 42 ℃/3 h.

### DAB staining and quantitation of relative ion leakage, MDA content, H_2_O_2_ content, and catalase activity

The HS-untreated and HS-treated petal disks were collected and vacuumized with DAB staining buffer to visualize ROS accumulation in petal disks. After 24 h in the dark, the infiltrated disks were submerged in ethanol to remove any pigment. Petal disks were harvested immediately after 12 h under HS to measure relative ion leakage (percentage) according to a previously reported method (Li et al. 2022; Wu et al. 2023). Briefly, 10 petal disks were vacuumized with 10 mL of deionized water until the disks completely sank to the bottom of the tube. The disks were then shaken at 28 ℃ and 200 rpm for 1 h, and the conductivity of each sample was measured and recorded as S1. After the measurement, tubes containing disks were put into boiling water for 20 min followed by shaking at 28 ℃ and 200 rpm for 2 h. The conductivity was measured again and recorded as S2. Deionized water was used as the control, and the conductivity was recorded as S0. Relative ion leakage was calculated as (S1-S0)/(S2-S0). To measure MDA content, four lily petal disks were added to 1 mL of a 10% trichloroacetic acid (TCA) solution, ground, and centrifuged to obtain the supernatant. Next, 0.2 mL of the supernatant was mixed with 0.2 mL of a 0.67% barbiturate solution and heated in boiling water for 15 min. After cooling, the absorbance was measured at 450 nm, 532 nm, and 600 nm. H_2_O_2_ content and catalase (CAT) activity were measured using the corresponding commercial kit according to the manufacturer’s instructions (D799773–0050 for H_2_O_2_, Sangon Biotech., China; A007 - 1–1 for CAT, Jiancheng Bioeng., China). Briefly, H_2_O_2_ reacts with titanium sulfate to produce a yellow titanium peroxide complex with a characteristic absorption at 415 nm. The activity of CAT was assayed by measuring the initial rate of disappearance of H_2_O_2_, and the decrease in H_2_O_2_ was monitored as a decrease in the optical density at 405 nm via UV spectrophotometry.

### Yeast one-hybrid assay

The upstream fragments of *HSFA3 A*, *WRKY33*, *CAT2*, and *GOLS1* were used separately for protein–promoter interactions. Promoter sequences were constructed into the pLacZi vector. Full-length and four different truncated fragments of LdHSFB2a were cloned into the pJG vector. The empty pJG and pLacZi vectors served as negative control. Transformation into yeast strain EGY48 was described by Yu et al. (2024). The transformants were selected by growing on deficient SD medium lacking Trp and Ura via a color change following the addition of X-gal (5-Bromo- 4-chloro- 3-indolyl β-D-galactopyranoside).

### Statistical analysis

Statistical Product Service Solutions v.17.0 (SPSS, USA) and GraphPad Prism 8.0.1 were used to analyze the experimental data and draw the diagrams. Student’s *t*-test was used to determine significance at *P* = 0.05. The Student–Newman–Keuls test (*P* < 0.05) was used to analyze variance.

## Supplementary Information

Supplementary Material 1. Supplemental Table S1. Primers used for *LdHSFB2a* ORF isolation in lily. Supplemental Table S2. Primers used for the quantitative real-time PCR in lily. Supplemental Table S3. Primers used for plasmid reconstruction.

## Data Availability

Not applicable.

## Abbreviations

ABA: Abscisic acid
APX: Ascorbate peroxidase
bp: Base pair
BSMV: The barley stripe mosaic
DAB: 3,3’-Diaminobenzidine
DBD DNA: Binding domain
GFP: Green fluorescent protein
HR-A/B: Heptad pattern of hydrophobic residues
HS: Heat stress
HSE: Heat stress element
HSF: Heat shock transcription factor
HSP: Heat shock protein
kD: Kilo Dalton
MDA: Malondialdehyde
MEGA: Molecular Evolutionary Genetics Analysis
NCBI: National Center for Biotechnology Information
NLS: Nuclear localization signal
OD: Optical density
ORF: Open reading frame
RFP: Red fluorescent protein
ROS: Reactive oxygen species
RT-qPCR: Real-time Quantitative Polymerase Chain Reaction
sHSPs: Small heat shock proteins
TAIR: Arabidopsis Information Resource
VIGS: Virus induced gene silencing
MDA: Malondialdehyde
CAT: Catalase
