# Supplementary material for: Molecular mechanism analysis of LdHSFB2a in lily thermotolerance
Source: Stress Biol. 2025 Jul 1;5(1):45. doi: 10.1007/s44154-025-00234-9 (PMC12209139; doi:10.1007/s44154-025-00234-9)
Supplement: Supplementary file 1 — Supplementary Material 1. Supplemental Table S1. Primers used for LdHSFB2a ORF isolation in lily. Supplemental Table S2. Primers used for the quantitative real-time PCR in lily. Supplemental Table S3. Primers used for plasmid reconstruction. [file 44154_2025_234_MOESM1_ESM.docx]

**Table Legends**

| **Supplemental Table S1.** Primers used for *LdHSFB2a* ORF isolation in lily. | | | |
| --- | --- | --- | --- |
| Gene name | Primer name | Sequence | Tm value |
| *LdHSFB2a* | HSFB2a-F | 5’-ATGAGAATTTCATTCCTCCAGGG-3’ | Tm = 57℃ |
| *LdHSFB2a* | HSFB2a-R | 5’-GTTCTGTGAAGTTGGTGGACGAA-3’ | Tm = 57℃ |

| **Supplemental Table S2.** Primers used for the quantitative real-time PCR in lily. | |
| --- | --- |
| Gene name | Sequence |
| *LdHSFB2a* | 5’-GGAGGTGAGGGGAGCAGGAATATT-3’ |
|  | 5’-ACGAAAGAAGTCACTACCCGCTCT-3’ |
| *18S rRNA* | 5’-AGTTGGTGGAGCGATTTGTCT-3’ |
|  | 5’-CCTGTTATTGCCTCAAACTTCC-3’ |
| *1391-GUS* | 5’-AGTTCTTTCGGCTTGTTG-3’ |
|  | 5’-TTCTACTTTACTGGCTTTGG-3’ |
| *HSFA1* | 5’-ATGGGAAGTGTCTATGTGGGG-3’ |
|  | 5’-CATTGATACTTGGCAGTTGTTGG-3’ |
| *HSFA2* | 5’-CAGACTGAGGTCGAGTTGGAAG-3’ |
|  | 5’-AACACAGCCCTCTTATCTTCTCG-3’ |
| *MBF1c* | 5’-GCTGATTAACGAGCGGGTGC-3’ |
|  | 5’-CACCCGCTCCATCTTCGCAAG-3’ |
| *HSFA3A* | 5’-CAGTTCACCTTATCCGCTGCGA-3’ |
|  | 5’-CTTAAAGTTCAGATGGTCGTGTCCTTG-3’ |
| *WRKY33* | 5’-CGGATGTTGCAGCAGAACCAAGGGATG-3’ |
|  | 5’-AGGCCAGCCATGAGTCTAGAAGCAAGT-3’ |
| *CAT2* | 5’-TGCTGGAAACTACCCTGAATGG-3’ |
|  | 5’-TCAACACCATACGTCCAACAGG-3’ |
| *GOLS1* | 5’-CAACGGCGACTATGTGAAGG-3’ |
|  | 5’-CCGGAGCTTGGAGTAGTTGA-3’ |
| *LlHSFB2a* | 5’-GTCGACCACATCATCTCATG-3’ |
| *Lilium longiflorum Thunb.* | 5’-AGCCCTTTTGACGATTGATC-3’ |
| *LlHSFB2a* | 5’-CTGAGTGCTTACGGTTTCAA-3’ |
| *Lilium longiflorum hybrid ‘WhiteHeaven’* | 5’-TCTCCCTCTTCAATTGCTCA-3’ |
| *LoHSFB2a* | 5’-CGAATGAAGAGCTTGTGTGA-3’ |
| *Lilium oriental hubrid ‘Siberia’* | 5’-CTCGGCTGAATCTCCAAATC-3’ |
| *LoHSFB2a* | 5’-ATATGCCCTCTTTCGTCTCA-3’ |
| *Lilium oriental hubrid ‘Sorbonne’* | 5’-ACACAGTGATCGTCAACAAG-3’ |

| **Supplemental Table S3.** Primers used for plasmid reconstruction. | | |
| --- | --- | --- |
| Empty vector | Vector name | Sequence |
| 1391-GUS | GUS-*proLdHSFB2a* | 5’-TTGGGCCCGGCGCGCCAAGCTTGGCTGCAGTTCTCTGATCTCACTCATCATACTTTC-3’ |
|  |  | 5’-GTGGACTCCTCTTAGAATTCCCGGGGATCCCGATGAACTTGATCTCAGCCTCGG-3’ |
| pCAMBIA1300-GFP | p1300-GFP-LdHSFB2a | 5’-AGAAAGCTTCTGCAGGGGCCCGGGGTCGACATGAGAATTTCATTCCTCCAGGG-3’ |
|  |  | 5’CAGCTCCTCGCCCTTGCTCACCATGGTACCGTTCTGTGAAGTTGGTGGACGAA-3’ |
| pGBKT7 | BD-LdHSFB2a | 5’-GACCTGCATATGGCCATGGAGGCCGAATTCATGAGAATTTCATTCCTCCAGGG-3’ |
|  |  | 5’-GTTATGCGGCCGCTGCAGGTCGACGGATCCTCAGTTCTGTGAAGTTGGTGGACGAA-3’ |
| pGBKT7 | BD-LdHSFB2a-VP16 | 5’-GACCTGCATATGGCCATGGAGGCCGAATTCATGAGAATTTCATTCCTCCAGGG-3’ |
|  |  | 5’-GCTGACATCGGTCGGGGGGGCACGGATCCCGTTCTGTGAAGTTGGTGGACGAA-3’ |
| pEAQ | pEAQ-LdHSFB2a | 5’-AGACAGTTGACTGTATCGCCGACCGGTATGAGAATTTCATTCCTCCAGGG-3’ |
|  |  | 5’-ATTTAATGAAACCAGAGTTAAAGGCCTTCAGTTCTGTGAAGTTGGTGGACGAA-3’ |
| pEAQ | pEAQ-LdHSFB2a-VP16 | 5’-AGACAGTTGACTGTATCGCCGACCGGTATGAGAATTTCATTCCTCCAGGG-3’ |
|  |  | 5’-ATTTAATGAAACCAGAGTTAAAGGCCTCTACCCACCGTACTCGTCAATTCC-3’ |
| pGreenII 62-SK | SK-LdHSFB2a | 5’-GAGCTCCACCGCGGTGGCGGCCGCTCTAGAATGAGA ATTTCATTCCTCCAGGG-3’  5’-GACGGTATCGATAAGCTTGATATCGAATTCCTAGTTC TGTGAAGTTGGTGGACGAA-3’ |
| pTRV2 | TRV2-LdHSFB2a | 5’-GATTCTGTGAGTAAGGTTACCGAATTCTCTAGAGTCG GCAGTAGACTCGAGGCGGAG-3’  5’-GCCCGGGCCTCGAGACGCGTGAGCTCGGTACCGTTCT GTGAAGTTGGTGGACGAA-3’ |
| pJG | pJG-LdHSFB2a | 5’-GATGTGCCAGATTATGCCTCTCCCGAATTCATGAGAATTTCATTCCTCCAGGG-3’ |
|  |  | 5’-CTCTGGCGAAGAAGTCCAAAGCTTCTCGAGCTAGTTCTGTGAAGTTGGTGGAC-3’ |
| pJG | pJG-LdHSFB2a-F1 | 5’-GATGTGCCAGATTATGCCTCTCCCGAATTCATGAGAATTTCATTCCTCCAGGGC-3’ |
|  |  | 5’-CTCTGGCGAAGAAGTCCAAAGCTTCTCGAGAAGGAGATTTTCCTGGCCCCTGCA-3’ |
| pJG | pJG-LdHSFB2a-F2 | 5’-GATGTGCCAGATTATGCCTCTCCCGAATTCTGCCGGATCAATCGTCAAAAGGGC-3’ |
|  |  | 5’-CTCTGGCGAAGAAGTCCAAAGCTTCTCGAGCATCATCTCTGTGCTGCTGCTTTTCA-3’ |
| pJG | pJG-LdHSFB2a-F3 | 5’-GATGTGCCAGATTATGCCTCTCCCGAATTCGAGGAAAATGAGCAATTGAAGAGG-3’ |
|  |  | 5’-CTCTGGCGAAGAAGTCCAAAGCTTCTCGAGATAGTTCGAGACAAGGTGCTGGAT-3’ |
| pJG | pJG-LdHSFB2a-F4 | 5’-GATGTGCCAGATTATGCCTCTCCCGAATTCAGCCTCGGTAGTGGTAGTGTCGGC-3’ |
|  |  | 5’-CTCTGGCGAAGAAGTCCAAAGCTTCTCGAGCTAGTTCTGTGAAGTTGGTGGACG-3’ |
| pLacZi | pz-*proHSFA3A* | 5’-ATCATTTCCTTTGATATTGGATCGGAATTCCTCTAGAGATTGAAGTTCGTAGTACAAA-3’ |
|  |  | 5’-TTTATATACATACAGAGCACATGCCTCGAGCGGTGTGGGCGGAGTGTTCGAGAA-3’ |
| pLacZi | pz-*proWRKY33* | 5’-ATCATTTCCTTTGATATTGGATCGGAATTCGGACGATTTTAGAGAAGAAGAAACA-3’ |
|  |  | 5’-TTTATATACATACAGAGCACATGCCTCGAGGAAAGTTATAACCGTTGATGATTCT |
| pLacZi | pz-*proCAT2* | 5’-ATCATTTCCTTTGATATTGGATCGGAATTCCGGCCGCACACAAGGAAGAGGAATG-3’ |
|  |  | 5’-TTTATATACATACAGAGCACATGCCTCGAGGAATCAAGTGTGTTCGGAGGATTGT-3’ |
| pLacZi | pz-*proGOLS1* | 5’-ATCATTTCCTTTGATATTGGATCGGAATTCAAAGAAACCGTCAAGTCGACGATTA-3’ |
|  |  | 5’-TTTATATACATACAGAGCACATGCCTCGAGTTATATTTCAAACTGTTTGACTACA-3’ |
